# Author Correction: Hsp70 and Hsp40 inhibit an inter-domain interaction necessary for transcriptional activity in the androgen receptor

**DOI:** 10.1038/s41467-019-12578-8

**Published:** 2019-10-07

**Authors:** Bahareh Eftekharzadeh, Varuna C. Banduseela, Giulio Chiesa, Paula Martínez-Cristóbal, Jennifer N. Rauch, Samir R. Nath, Daniel M. C. Schwarz, Hao Shao, Marta Marin-Argany, Claudio Di Sanza, Elisa Giorgetti, Zhigang Yu, Roberta Pierattelli, Isabella C. Felli, Isabelle Brun-Heath, Jesús García, Ángel R. Nebreda, Jason E. Gestwicki, Andrew P. Lieberman, Xavier Salvatella

**Affiliations:** 1 Institute for Research in Biomedicine (IRB Barcelona), The Barcelona Institute of Science and Technology, Baldiri Reixac 10, 08028 Barcelona, Spain; 2 Joint BSC-IRB Research Programme in Computational Biology, Baldiri Reixac 10, 08028 Barcelona, Spain; 3 Department of Pathology, University of Michigan, Ann Arbor, MI 48109 USA; 4 University of California at San Francisco, Department of Pharmaceutical Chemistry, 675 Nelson Rising Lane, San Francisco, CA 94158 USA; 5 CERM and Department of Chemistry “Ugo Schiff”, University of Florence, Via Luigi Sacconi 6, 50019 Sesto Fiorentino, Florence, Italy; 6 ICREA, Passeig Lluís Companys 23, 08010 Barcelona, Spain

**Keywords:** Chaperones, Neuromuscular disease, Solution-state NMR, Nuclear receptors

Correction to: *Nature Communications* 10.1038/s41467-019-11594-y, published online 8 August 2019.

The original version of this Article contained an error in the spelling of the author Roberta Pierattelli, which was incorrectly given as Roberta Pieratelli. This has now been corrected in both the PDF and HTML versions of the Article.

