# Supplementary material for: Hsp70 and Hsp40 inhibit an inter-domain interaction necessary for transcriptional activity in the androgen receptor
Source: Nat Commun. 2019 Aug 8;10:3562. doi: 10.1038/s41467-019-11594-y (PMC6687723; doi:10.1038/s41467-019-11594-y)
Supplement: Supplementary file 1 — Supplementary Information [file 41467_2019_11594_MOESM1_ESM.pdf]

Hsp70 and Hsp40 inhibit an inter-domain interaction necessary for transcriptional activity in the androgen receptor

Eftekharzadeh, Banduseela, Chiesa, Martínez-Cristóbal et al

Supplementary figures

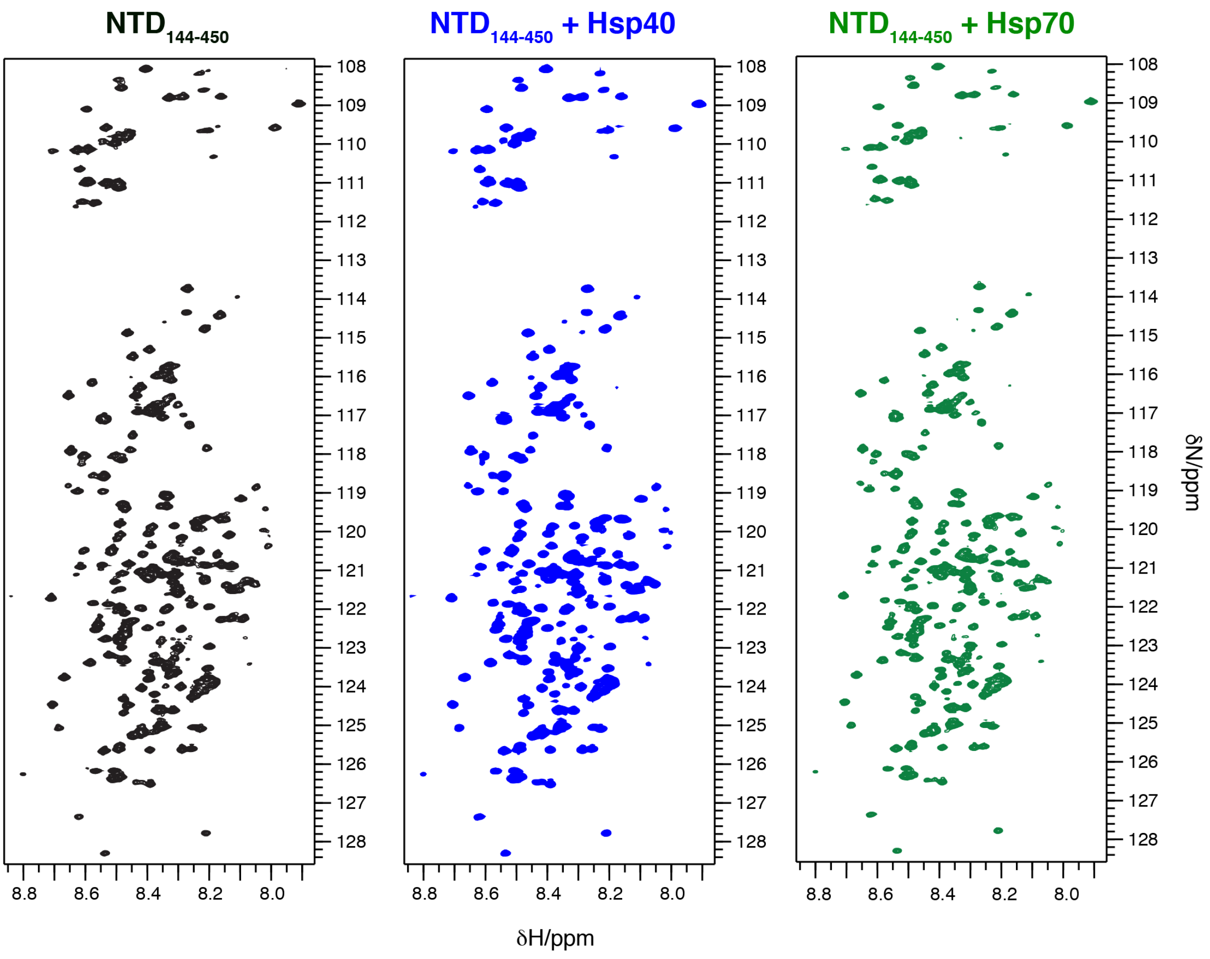

Supplementary Figure 1 - Hsp40 and Hsp70 do not interact with $NTD_{144\text{-}450}$.

$^{1}H,^{15}N$ HSQC spectra of 33 μM $NTD_{144\text{-}450}$ in the absence (black) and in the presence of 1 molar equivalent of Hsp40 (blue) and Hsp70 (green) under the solution conditions used in Figure 1.

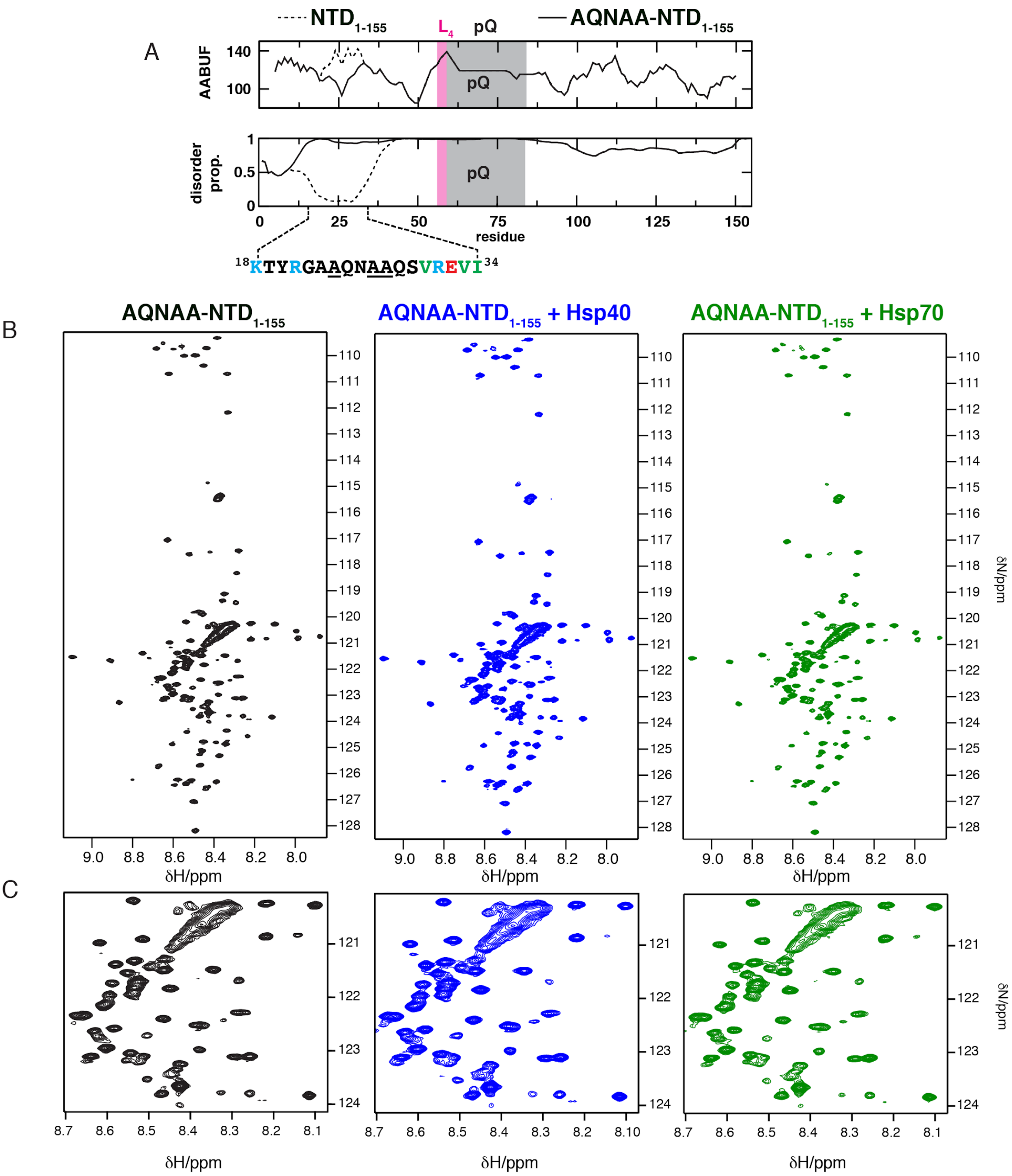

Supplementary Figure 2 - AQNAA-NTD_{1-155} has a decreased affinity for Hsp40 and Hsp70

A) Change in accessible area buried upon folding (AABUF), and in disorder propensity, predicted by the PONDR algorithm[1], caused by the mutations introduced in the binding motif of these molecular chaperones. In the sequence of the motif the positively charged residues are shown in blue, negatively charged residues are shown in red, the hydrophobic residues are shown in green and the residues underlined correspond to those mutated.

B and C) Spectra of 33 μM AQNAA-NTD_{1-155} in the absence and in the presence of 1 molar equivalent of Hsp40 (blue) and Hsp70 (green).

A

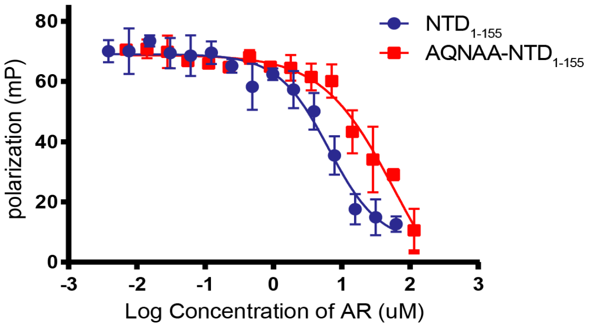

B

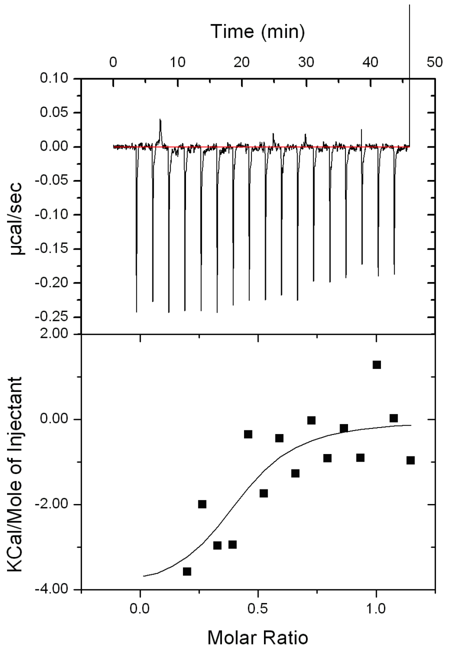

Supplementary Figure 3 - AQNAA-NTD_{1-155} has decreased affinity for Hsp70 and Hsp40 compared to wild type NTD_{1-155}.

A) Competition FP experiments, in which the relative affinity of NTD_{1-155} and AQNAA-NTD_{1-155} were compared by competition with FAM-NRLLLTG binding to $Hsp70_{SBD}$. See Figure 2 and Methods for details. Briefly, 12 µM Hsp70 SBD (residues 394-509) and 25 nM FAM-NRLLLTG were incubated with varying concentrations of AR protein for 30 minutes at room temperature in assay buffer (100 mM Tris, 20 mM KCl, 6 mM MgCl2, 0.001% Triton-X 100, pH 7.4). After incubation, FP was measured (excitation: 485 nm emission: 535 nm) using a SpectraMax M5 plate reader. Results are the average of experiments performed in triplicate and the error bars represent S.E.M.

B) Hsp40 binds NTD_{1-155}, as measured by ITC. See Figure 2 for methods. Kd values were calculated as the average of two independent experiments ± S.E.M.

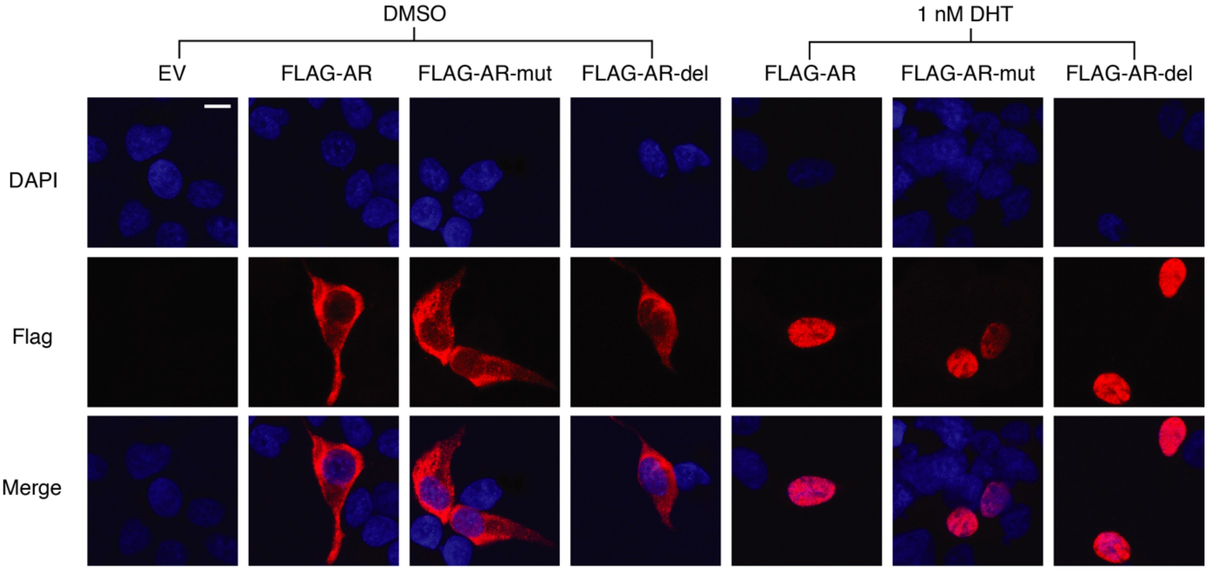

Supplementary Figure 4 - Activation of AR by DHT causes nuclear translocation.

Subcellular localization, as obtained by immunofluorescence, of FLAG-AR, FLAG-AR-mut and FLAG-AR-del in transfected HEK293T cells in the absence and presence of 1nM DHT (n = 3). The scale bar represents 10 μm.

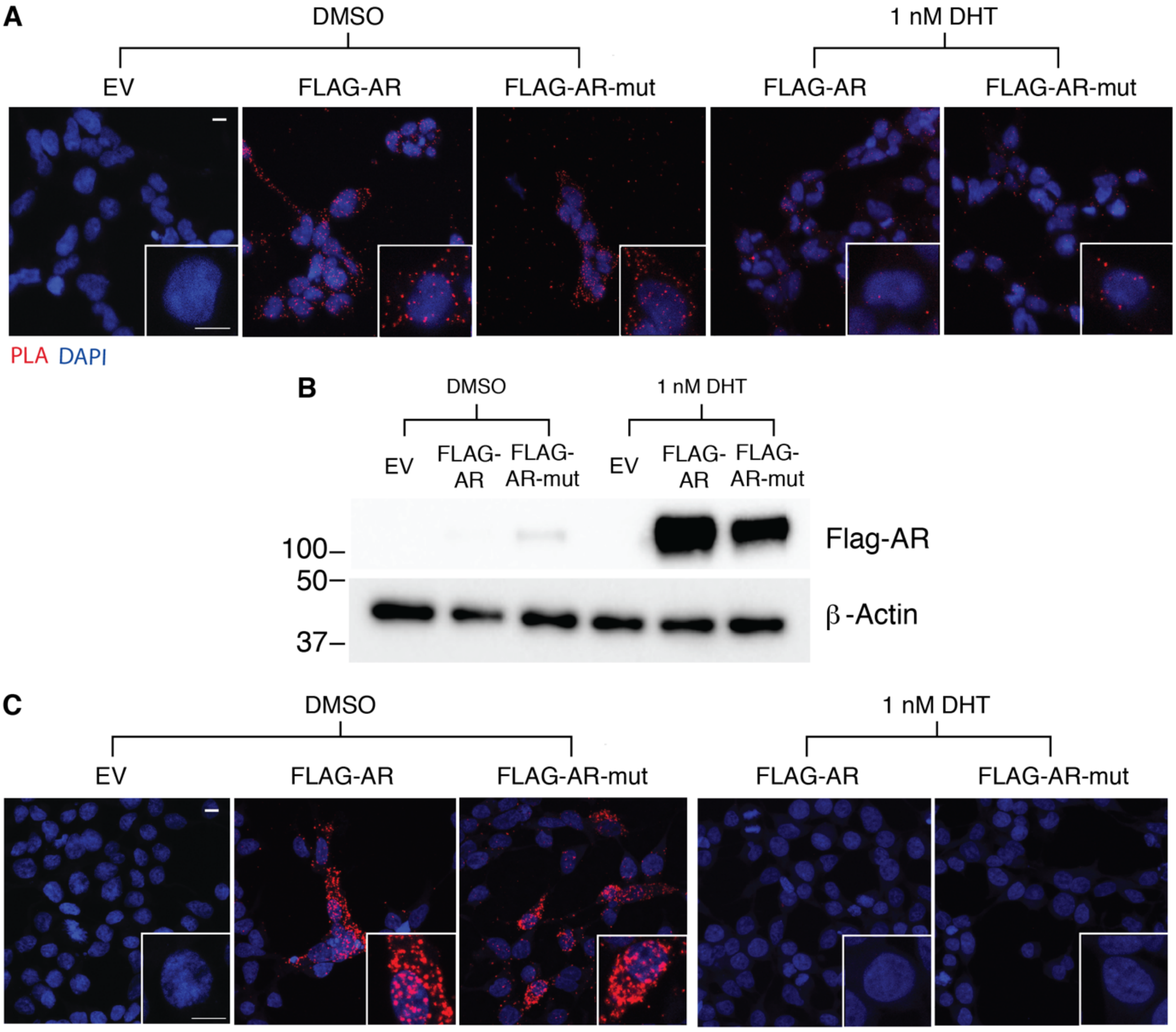

Supplementary Figure 5 - Interaction between FLAG-AR-mut and Hsp40 and Hsp70.

A) The interaction between FLAG-AR as well as FLAG-AR-mut with endogenous Hsp70 in the absence or presence of 1 nM DHT was studied in HEK293T cells by PLA assay. EV stands for empty vector, FLAG-AR/Hsp70 complexes appear as red dots and DAPI indicates the localization of nuclei. The scale bars represent 10 μm.

B) Analysis by Western blotting of the levels of FLAG-AR and FLAG-AR-mut in transfected HEK293T cells in the absence or presence of 1 nM DHT. β-actin immunoblot is shown as a loading control.

C) Assay equivalent to that presented in panel A to study the interaction with the endogenous Hsp40. The scale bars represent 10 μm.

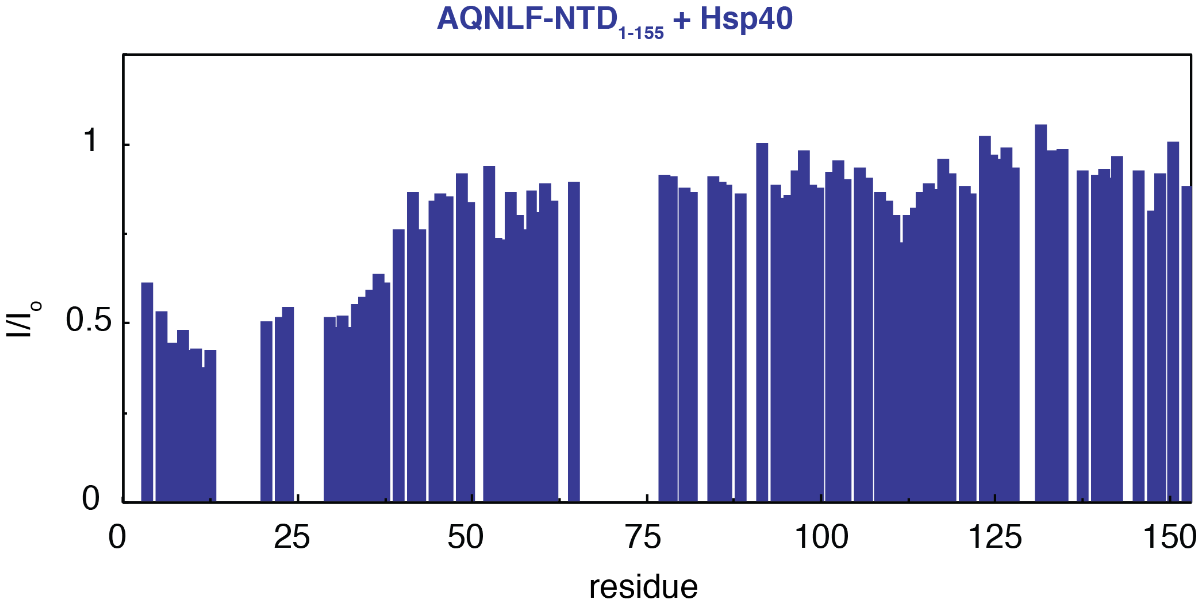

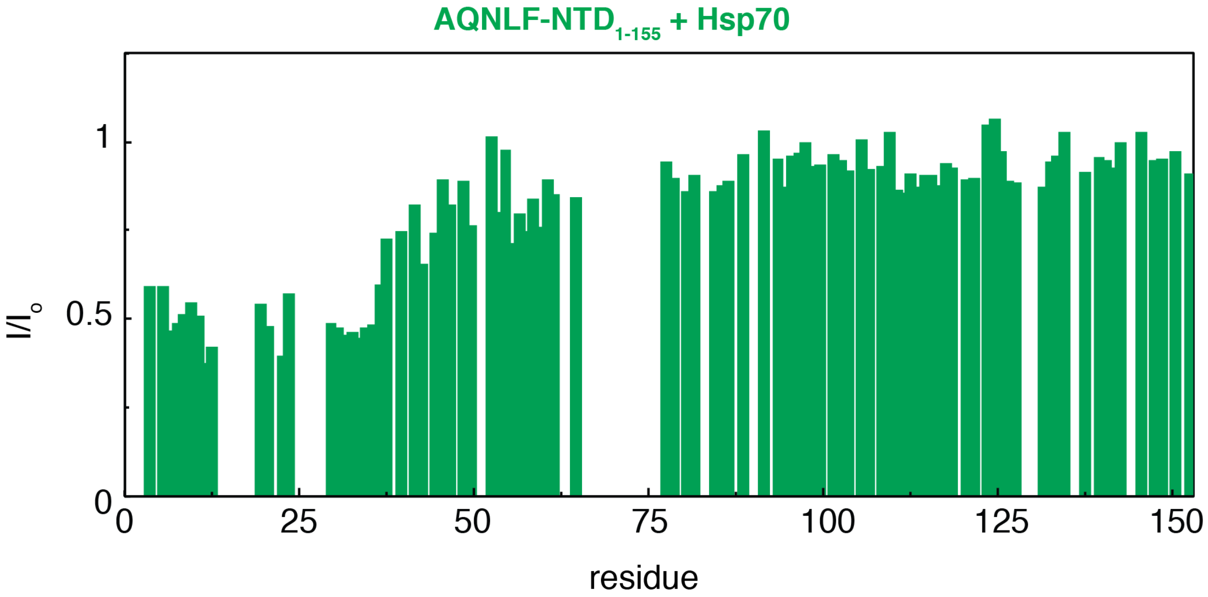

Supplementary Figure 6 - AQNLF-NTD$_{1-155}$ has high affinity for Hsp40 and Hsp70

Changes in the intensity of the resonances of 33 μM AQNLF-NTD$_{1-155}$ caused by 1 molar equivalents of Hsp40 (top) and Hsp70 (bottom)

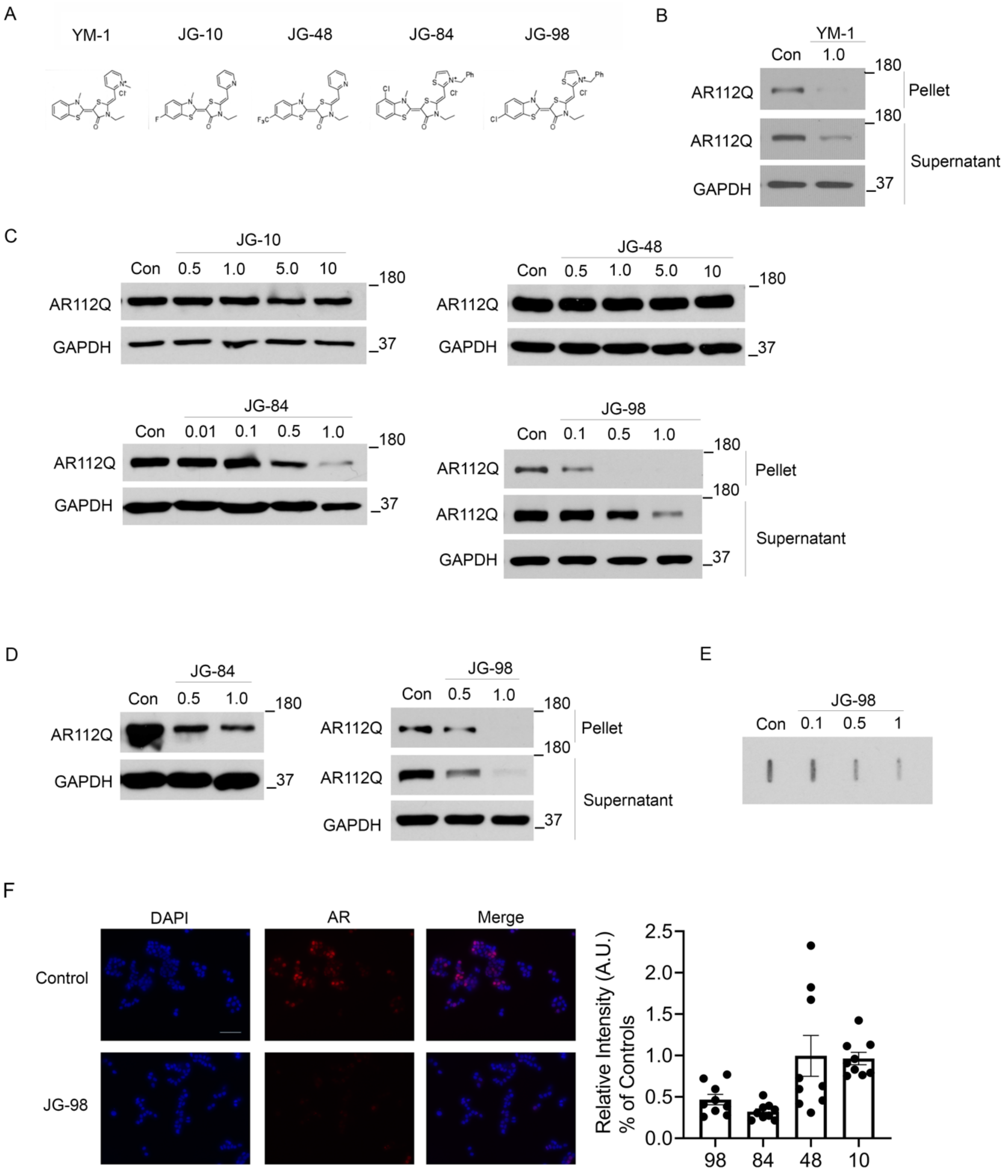

Supplementary Figure 7 - Effects of JG series compounds on AR112Q clearance.

A) Structures of YM-1 and its derivatives JG-10, JG-48, JG-84 and JG98.

B) YM-1 promotes AR 112Q clearance in PC12 cells. PC12 cells were induced to express AR112Q in the presence of R1881 (10nM) for 48 hr, washed to remove doxycycline to turn off the transgene, and then incubated for 72 hr in the presence or absence of 1 μM YM-1.

Lysates were separated in to supernatant and pellet by 15000 g centrifugation and analyzed by western blot.

C) PC12 cells were induced to express AR112Q in the presence of R1881 (10nM) and the indicated concentrations (μM) of JG-10, JG-48, JG-84 and JG-98 for 48 h. Cell lysates were analyzed as in panel B. Shown are supernatant fractions unless otherwise indicated. Con=vehicle control.

D) PC12 cells were induced to express AR112Q in the presence of R1881 (10nM) for 48 hr, washed to remove doxycycline to turn off the transgene, and then incubated for 72 hr in the presence or absence of the indicated concentrations of JG-84 or JG-98. Cell lysates were analyzed as in panel B. Shown are supernatant fractions unless otherwise indicated.

E) PC12 cells were induced to express AR112Q in the presence of R1881 (10nM) for 48 hr in the presence of indicated concentrations (μM) of JG-98. Lysates were analyzed by filter trap assay. Con=vehicle control.

F) Left: PC12 cells were induced to express AR112Q in the presence of R1881 (10nM) for 48 hr, washed to remove doxycycline to turn off the transgene, then incubated for 72 hr with 1 μM JG-98 or vehicle. Cells were fixed and stained for AR. Nuclei were stained by DAPI. Scale bar, 50 μm. Right: Quantification of AR fluorescence within nuclei after treatment with 1 mM JG-10, JG-48, JG-84 or JG-98. Data are mean ± S.E.M from 3 independent experiments. Values are relative to vehicle control.

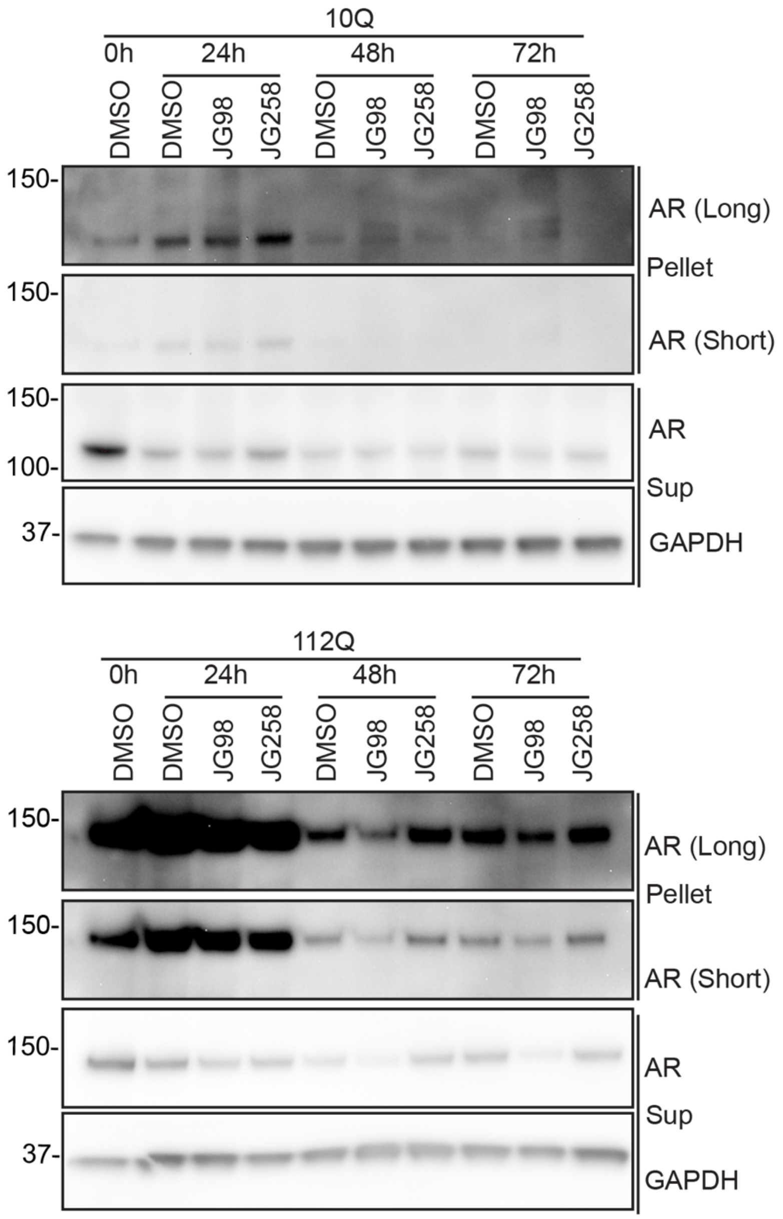

Supplementary Figure 8 – JG-98 promotes clearance of soluble and aggregated polyQ AR.

PC12 cells were induced to express wild type (AR10Q) or polyQ AR (AR112Q) in the presence of R1881 (10nM) for 48 hr, washed to remove doxycycline to turn off the transgene, and then incubated for 24, 48, or 72 hr in the presence or absence of DMSO, 1 μM JG-98, or 1 μM JG-258. The amount of AR in the soluble and insoluble fractions was analyzed by western blot.

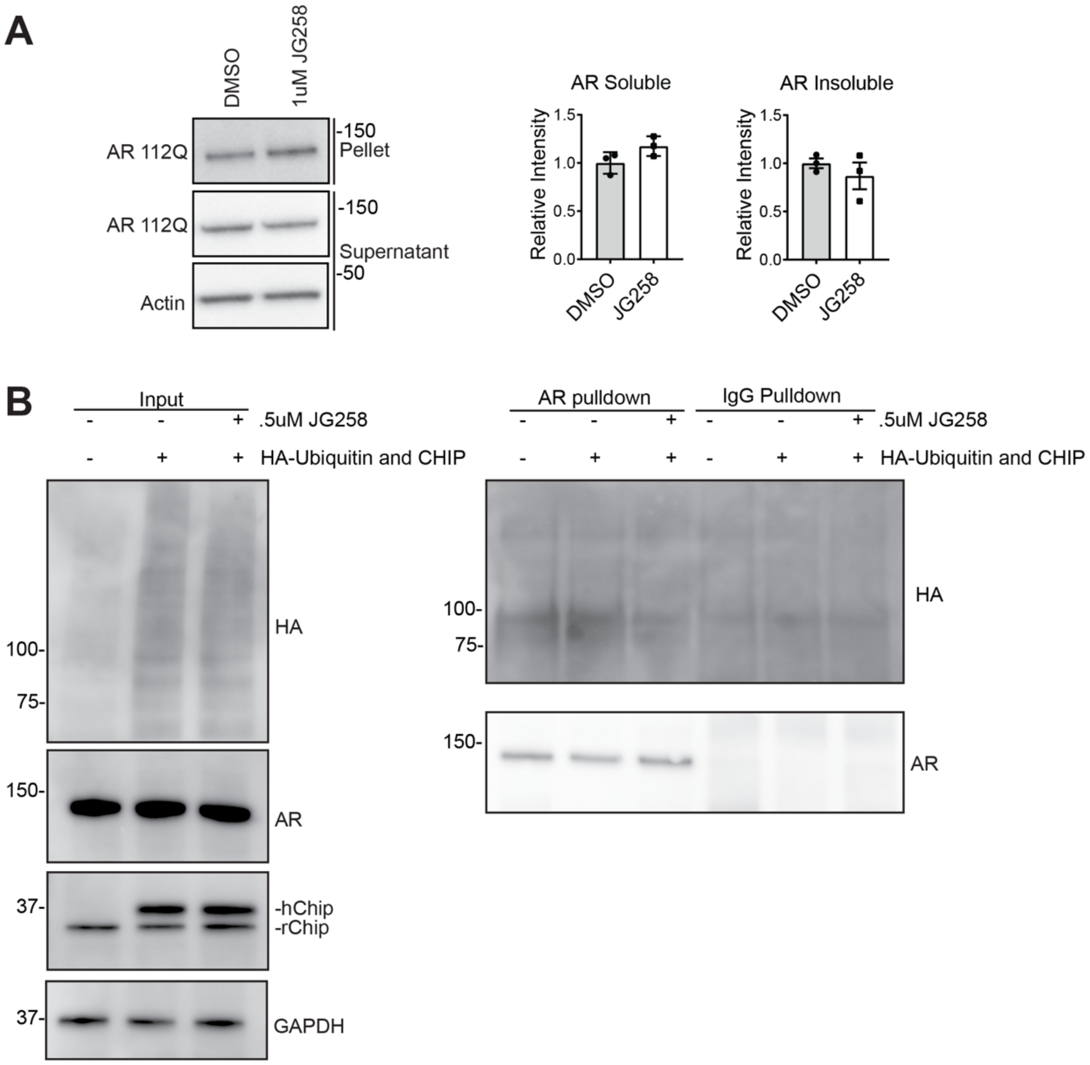

Supplementary Figure 9 – JG-258 does not alter polyQ AR levels or ubiquitination.

A) PC12 cells were induced to express AR112Q in the presence of R1881 (10nM) and DMSO or 1μM JG-258 for 48 h. Cell lysates were analyzed by western blot for soluble and insoluble AR. Quantified at right. Data are mean ± S.E.M from n=3 experiments.

B) PC12 cells were transiently transfected to express HA-ubiquitin and human CHIP (hCHIP), and AR112Q expression was induced for 48 hr. Cells were treated with 0.5 μM JG-258 for the last 24 hrs and with 10 μM MG132 for the last 16 hrs. AR112Q was immunoprecipitated from lysates and then probed for ubiquitin (HA). Left: input. Right: pull down.

Supplementary reference

1. Romero, P. *et al.* Sequence complexity of disordered protein. *Proteins: Struct. Funct. Bioinf.* **42**, 38–48 (2001).
