# Supplementary material for: Hsp70 and Hsp40 inhibit an inter-domain interaction necessary for transcriptional activity in the androgen receptor
Source: Nat Commun. 2019 Aug 8;10:3562. doi: 10.1038/s41467-019-11594-y (PMC6687723; doi:10.1038/s41467-019-11594-y)
Supplement: Supplementary file 3 — Reporting Summary [file 41467_2019_11594_MOESM3_ESM.pdf]

nature research

Corresponding author(s): Jason E. Gestwicki, Andrew P. Lieberman and Xavier. Salvatella

Last updated by author(s): 2019-7-23

Reporting Summary

Nature Research wishes to improve the reproducibility of the work that we publish. This form provides structure for consistency and transparency in reporting. For further information on Nature Research policies, see Authors & Referees and the Editorial Policy Checklist.

Statistics

For all statistical analyses, confirm that the following items are present in the figure legend, table legend, main text, or Methods section.

n/a	Confirmed	
☐	☒	The exact sample size (*n*) for each experimental group/condition, given as a discrete number and unit of measurement
☒	☐	A statement on whether measurements were taken from distinct samples or whether the same sample was measured repeatedly
☐	☒	The statistical test(s) used AND whether they are one- or two-sided<br>*Only common tests should be described solely by name; describe more complex techniques in the Methods section.*
☒	☐	A description of all covariates tested
☐	☒	A description of any assumptions or corrections, such as tests of normality and adjustment for multiple comparisons
☐	☒	A full description of the statistical parameters including central tendency (e.g. means) or other basic estimates (e.g. regression coefficient) AND variation (e.g. standard deviation) or associated estimates of uncertainty (e.g. confidence intervals)
☐	☐	For null hypothesis testing, the test statistic (e.g. *F*, *t*, *r*) with confidence intervals, effect sizes, degrees of freedom and *P* value noted<br>*Give P values as exact values whenever suitable.*
☐	☐	For Bayesian analysis, information on the choice of priors and Markov chain Monte Carlo settings
☐	☐	For hierarchical and complex designs, identification of the appropriate level for tests and full reporting of outcomes
☐	☐	Estimates of effect sizes (e.g. Cohen's *d*, Pearson's *r*), indicating how they were calculated

Our web collection on statistics for biologists contains articles on many of the points above.

Software and code

Policy information about availability of computer code

Data collection	Data collection was carried out by using the software provided by the supplier of the apparatus.
Data analysis	ITC data was processed in Origin 7.0. FP data were fit in GraphPad PRISM. NMR data was processed with NMRPipe and analyzed with CCPN analysis.

For manuscripts utilizing custom algorithms or software that are central to the research but not yet described in published literature, software must be made available to editors/reviewers. We strongly encourage code deposition in a community repository (e.g. GitHub). See the Nature Research guidelines for submitting code & software for further information.

Data

Policy information about availability of data

All manuscripts must include a data availability statement. This statement should provide the following information, where applicable:

- Accession codes, unique identifiers, or web links for publicly available datasets
- A list of figures that have associated raw data
- A description of any restrictions on data availability

FP, ITC and NMR data available from the corresponding authors on request.

Field-specific reporting

Please select the one below that is the best fit for your research. If you are not sure, read the appropriate sections before making your selection.

☒ Life sciences ☐ Behavioural & social sciences ☐ Ecological, evolutionary & environmental sciences

Life sciences study design

All studies must disclose on these points even when the disclosure is negative.

Sample size	Experiments were done with independent biological samples (usually n≥3) and highly comparable results were obtained. This sample size is widely accepted as sufficient when using independent biological samples.
Data exclusions	No data was excluded
Replication	All attempts at replication were successful in ≥3 experiments with independent biological samples. The methods and reagents used are described in detail, so that replication of the experiments by other scientists should be facilitated
Randomization	Samples were organized by experimental variable, measurements were done in an automated manner (e.g. by the microscope) and the results are reported in completion.
Blinding	Not applicable.

Reporting for specific materials, systems and methods

We require information from authors about some types of materials, experimental systems and methods used in many studies. Here, indicate whether each material, system or method listed is relevant to your study. If you are not sure if a list item applies to your research, read the appropriate section before selecting a response.

Materials & experimental systems

n/a	Involved in the study	
☐	☒	Antibodies
☐	☒	Eukaryotic cell lines
☒	☐	Palaeontology
☒	☐	Animals and other organisms
☒	☐	Human research participants
☒	☐	Clinical data

Methods

n/a	Involved in the study	
☒	☐	ChIP-seq
☒	☐	Flow cytometry
☒	☐	MRI-based neuroimaging

Antibodies

Antibodies used	The data are provided in the manuscript. For Western Blot analyses the following antibodies were used: anti-beta-actin-HRP (ab8224) (Abcam), rabbit-anti-FLAG (M2, F7425) (Sigma), mouse-anti-HSP70/72 (ADI-SPA-810) and mouse-anti-HSP40 (ADI-SPA-400-D) (Enzo Life Science). Additional primary antibodies were against Akt (C6E7; Cell Signaling Technology), AR (N-20; Santa Cruz Biotechnology), ERK1/2 (Cell Signaling Technology), GAPDH (NB-600-502; Novus Biologicals), HSP90 (H114, sc7947; Santa Cruz Biotechnology), CHIP (Thermo Scientific), HA (Covance), HSP25 (ADI-SPA-801-488; Enzo Life Science), and HSP70 (ADI-SPA-812; Enzo Life Science). HRP conjugated secondary antibodies were from Bio-Rad. AlexaFluor conjugated secondary antibodies were from Life Technologies. Mounting medium with DAPI (H-1200) was from Vector Labs. For immunoprecipitation, pull down was performed with protein A–agarose beads (Santa Cruz Biotechnology) and the anti-AR antibody (PG21; Millipore).
Validation	The data are provided in the manuscript

Eukaryotic cell lines

Policy information about cell lines

Cell line source(s)	HEK293T cells were from Xavier Salvatella's lab aliquots (originally obtained from ATCC). PC12 cells expressing tet-inducible AR112Q were gift of Diane Merry (Thomas Jefferson University).
Authentication	Cells initially obtained from ATCC had been authenticated by the supplier (morphology, karyotyping, PCR) and PC12 cells were authenticated by WB and IF for AR expression.
Mycoplasma contamination	No mycoplasma contamination was confirmed by the authors.
Commonly misidentified lines (See ICLAC register)	No commonly misidentified cell lines were used
