# Supplementary material for: Hsp70 and Hsp40 inhibit an inter-domain interaction necessary for transcriptional activity in the androgen receptor
Source: Nat Commun. 2019 Aug 8;10:3562. doi: 10.1038/s41467-019-11594-y (PMC6687723; doi:10.1038/s41467-019-11594-y)

Figure 3B

FLAG

150
100

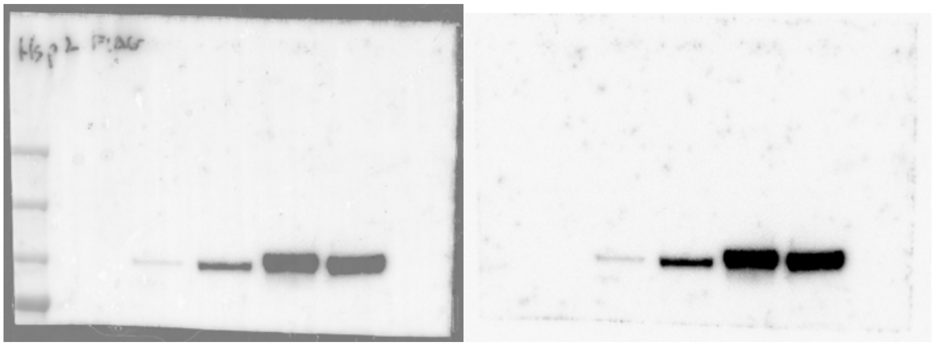

Actin

50
37

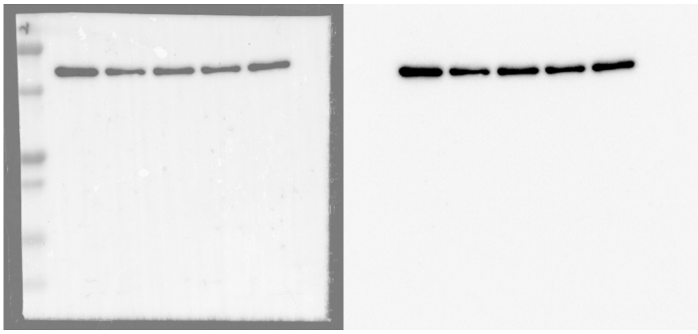

Figure 5A

Cropped gels.

AR

180

115

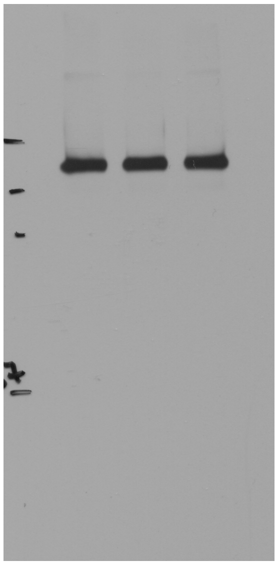

CHIP

37

25

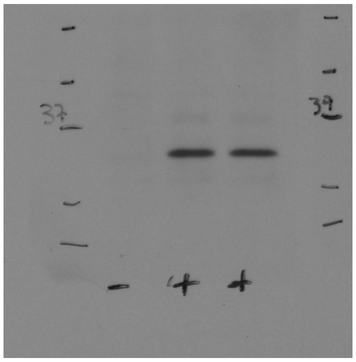

GAPDH

37

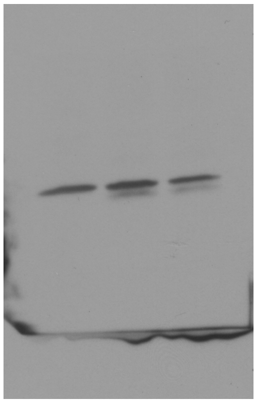

HA

180

115

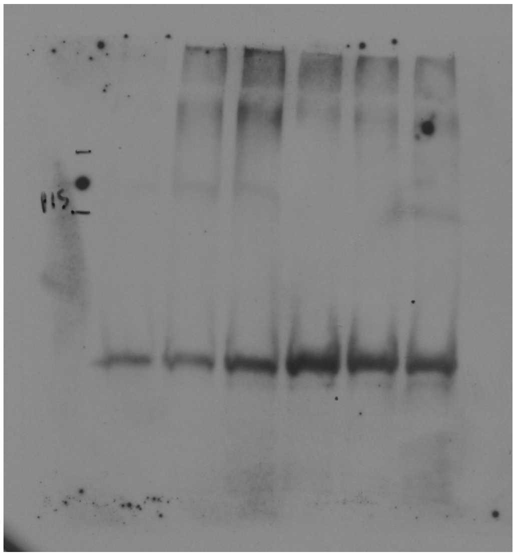

Other images are from cut blots and entire image is shown in Figure

Figure 5B

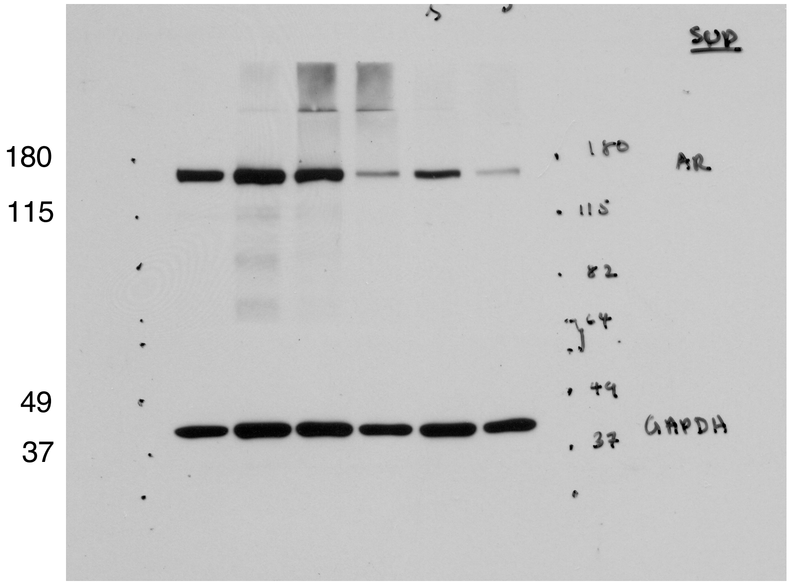

Figure 5C

Akt (top)

GAPDH (bottom)

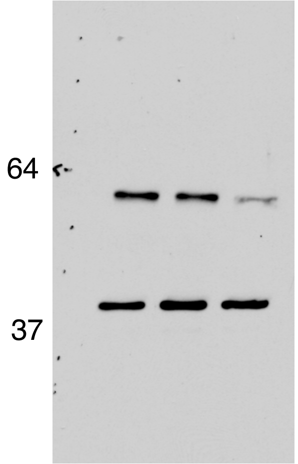

ERK

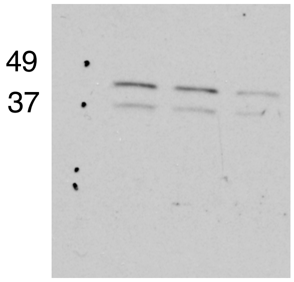

Hsp25 (lower band)

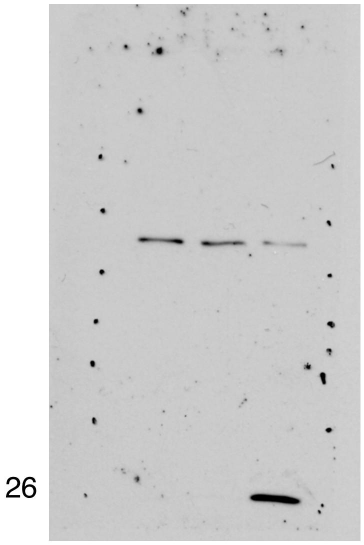

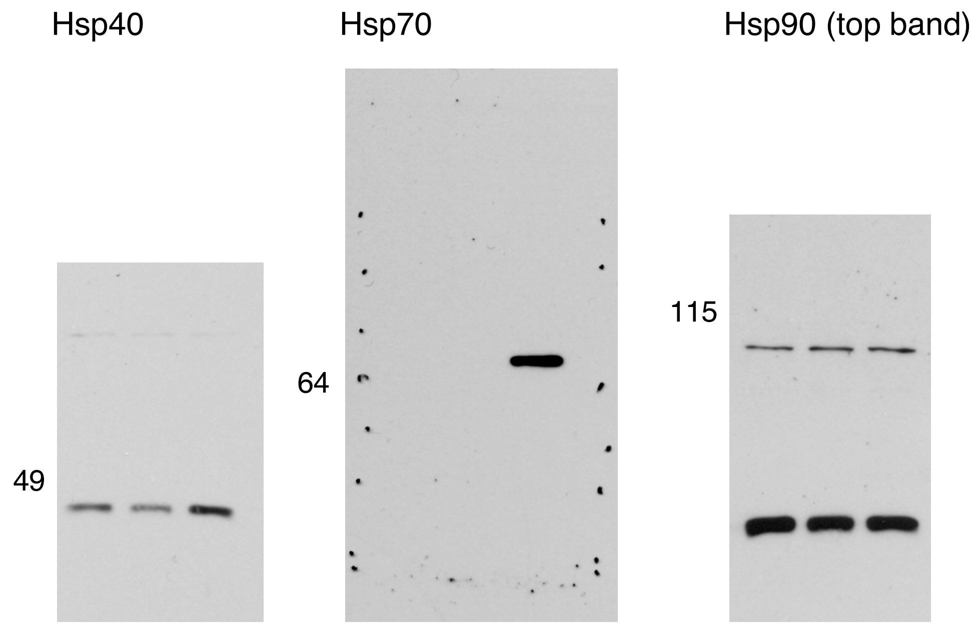

Figure 5D

Entire images shown in figure

Figure 6B

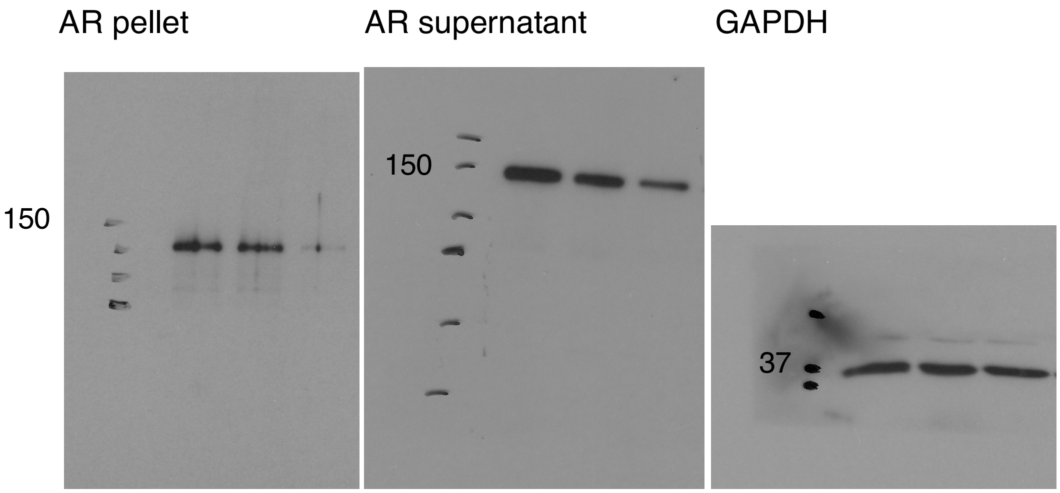

Figure 7A

AR

150

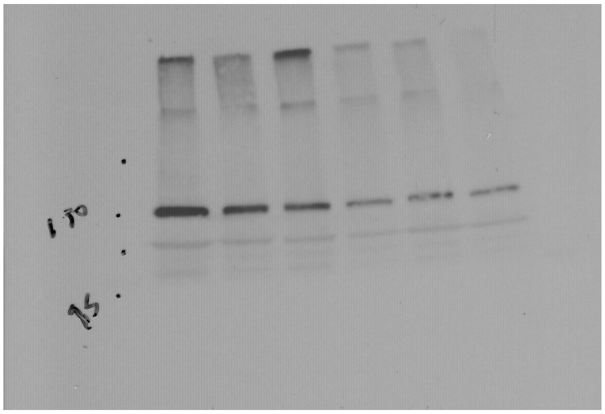

GAPDH

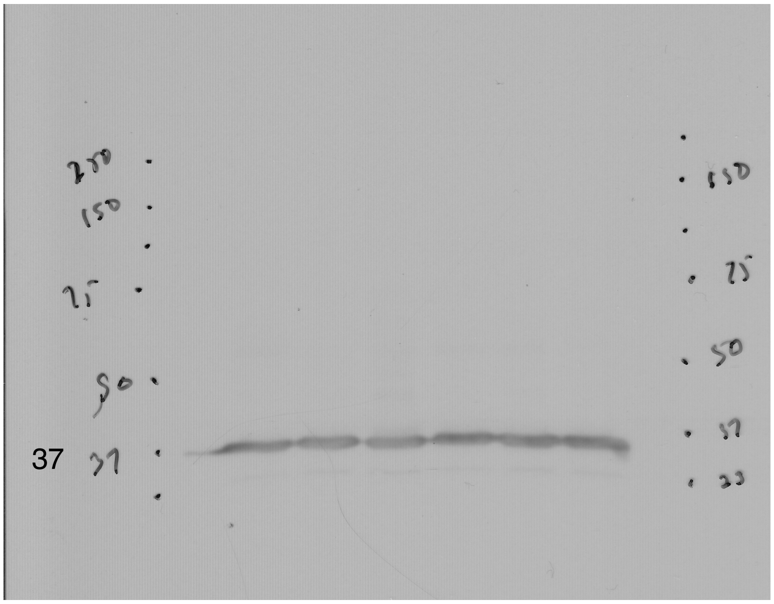

Figure 7D

Entire image shown in figure

Figure 7F

Images are from cut blots and entire image is shown in figure

Supplementary Figure 5B

FLAG

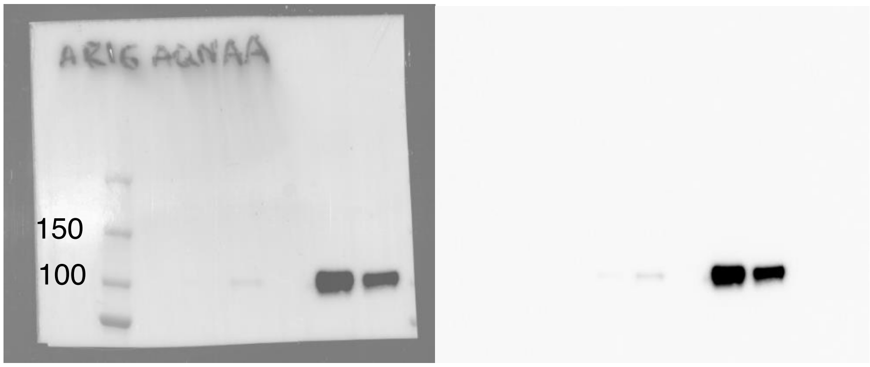

Actin

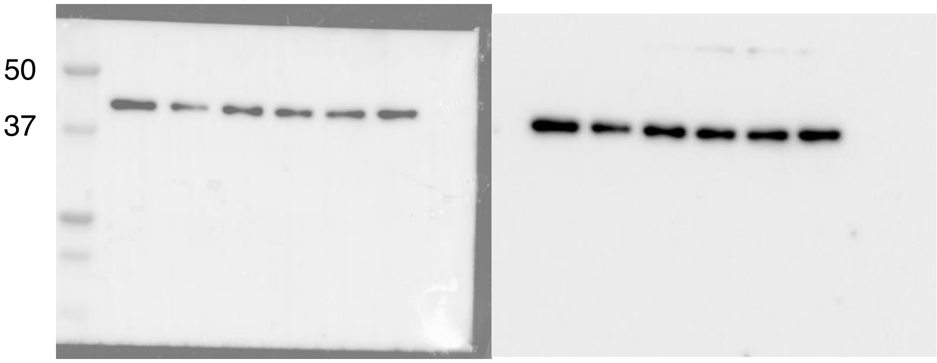

Supplementary Figure 7B

AR sup (top)

GAPDH (bottom)

AR pellet

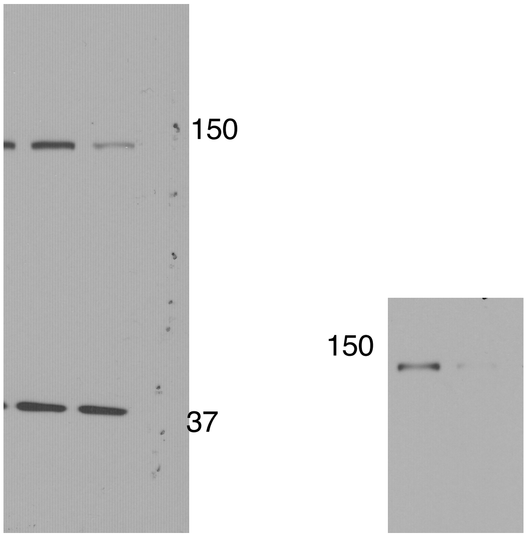

Supplementary Figure 7C

AR (top)

GAPDH (bottom; JG-10)

180
115
49
37

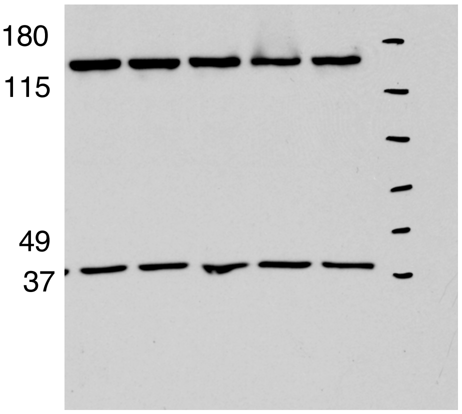

AR (top)

GAPDH (bottom, JG-48)

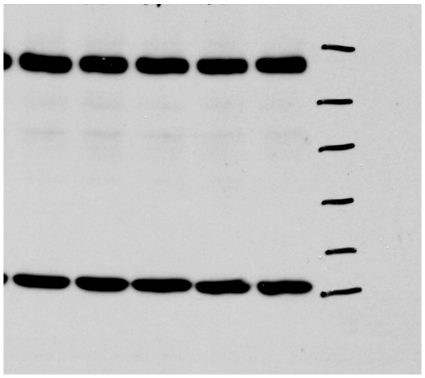

180
115
49
37

AR (top)

GAPDH (bottom; JG-84)

180
115
49
37

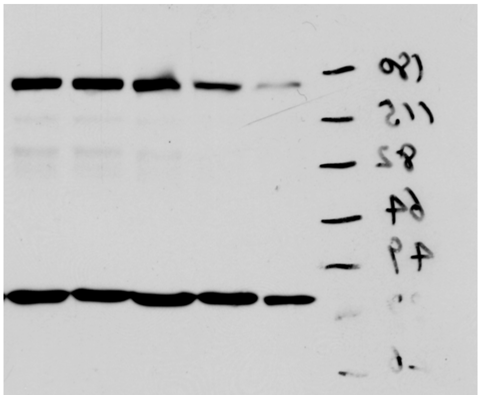

AR pellet (top, JG-98)

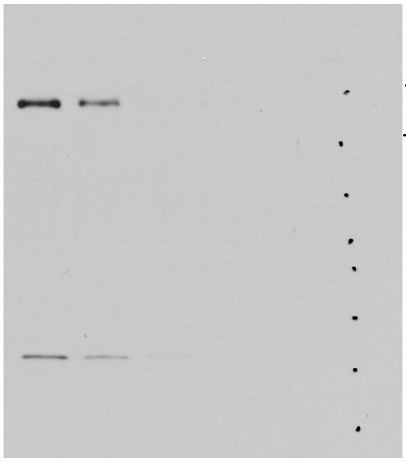

180
115

AR supernatant (top)

GAPDH (bottom, JG-98)

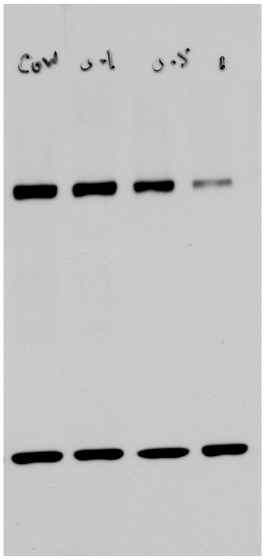

180

115

37

Supplementary Figure 7D

AR (top);

GAPDH (bottom, JG-84)

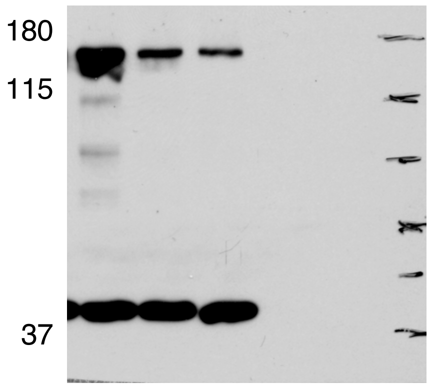

AR pellet

(top, JG-98)

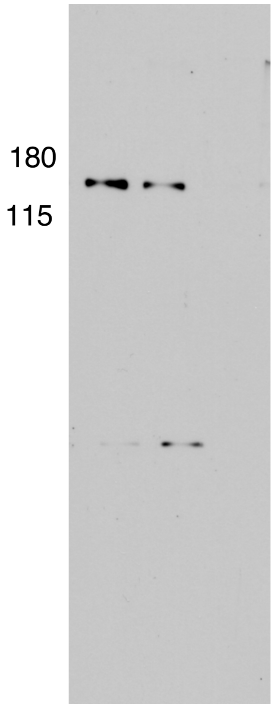

AR supernatant (top);

GAPDH (bottom, JG-98)

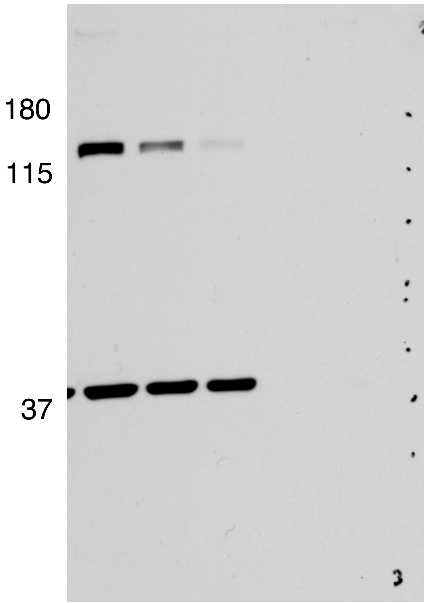

Supplementary Figure 7E

Entire image shown in figure

Supplementary Figure 8

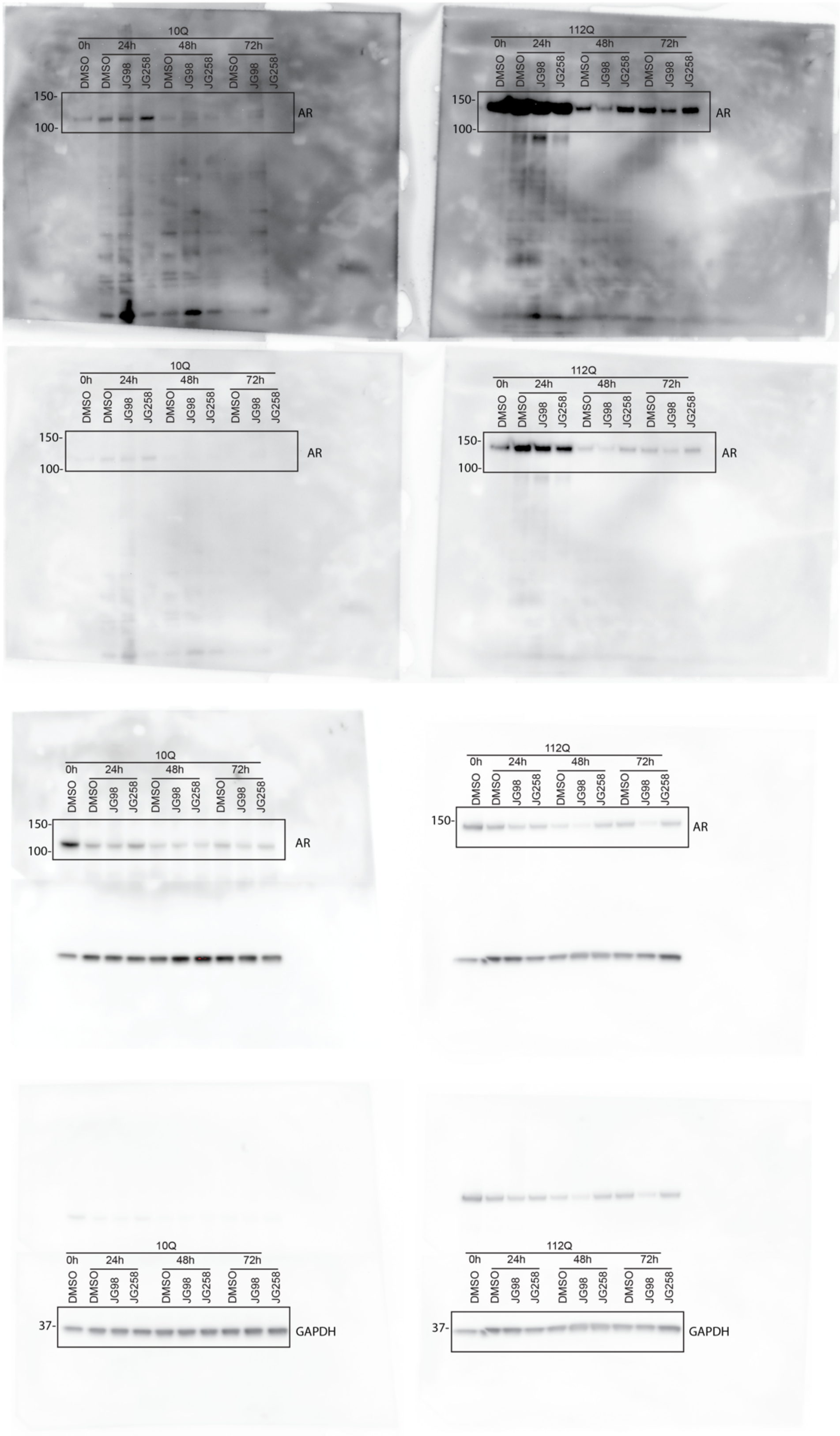

Supplementary Figure 9A

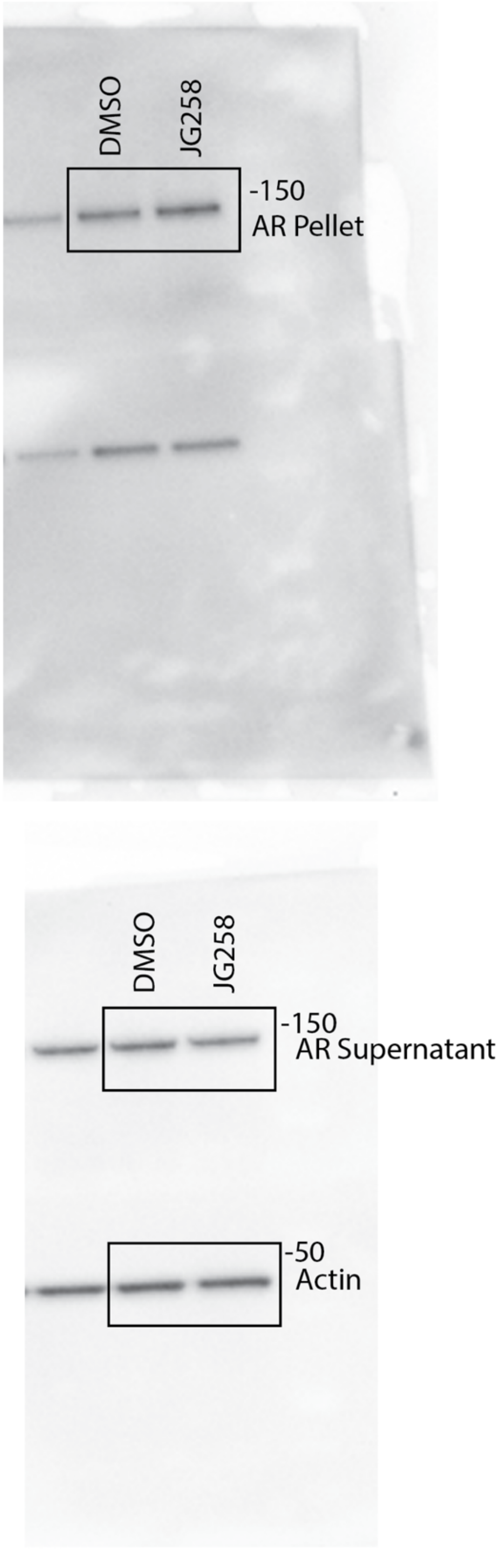

Supplementary Figure 9B

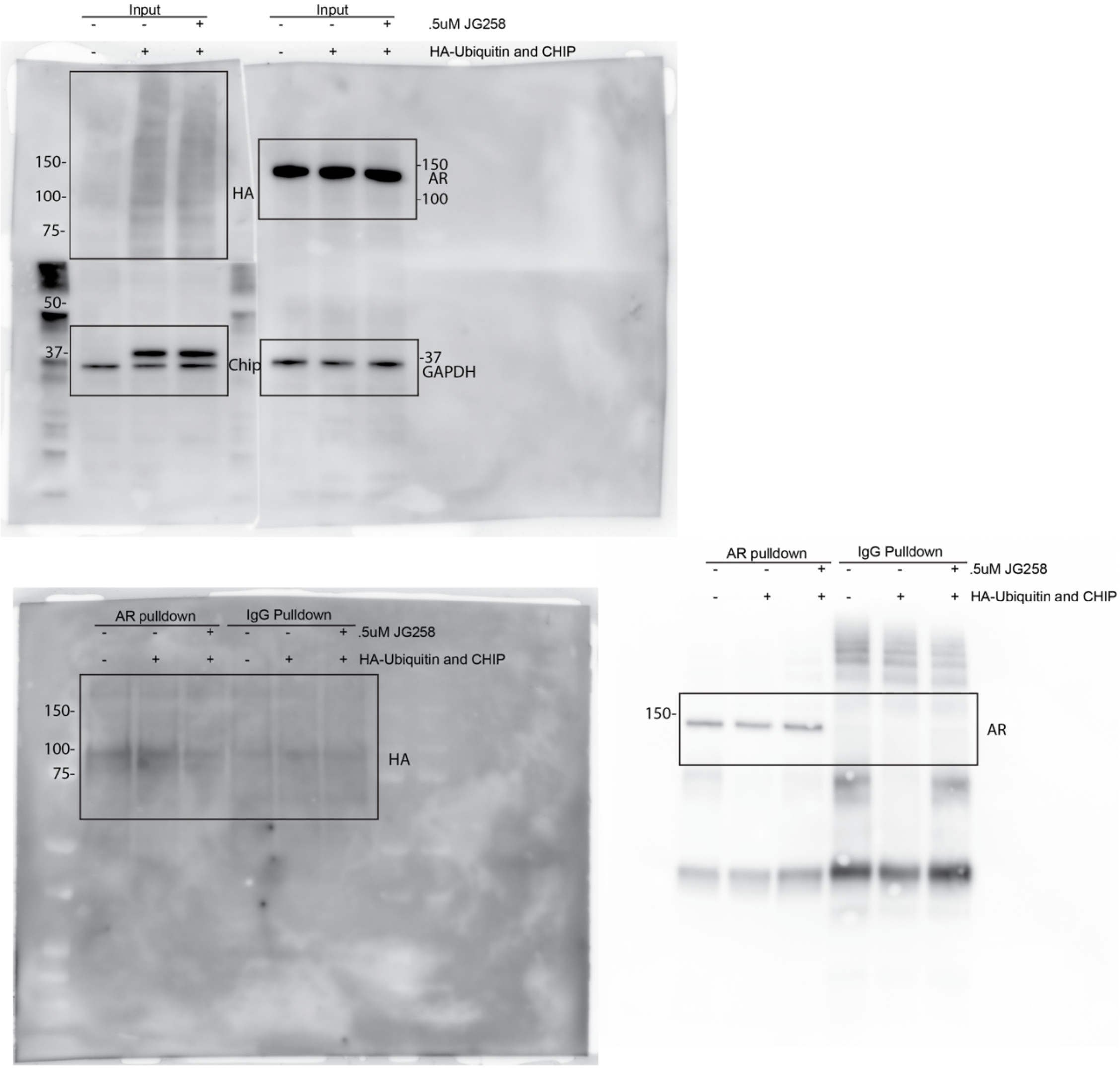

Supplement: Supplementary file 4 — Source Data [file 41467_2019_11594_MOESM4_ESM.zip › Uncropped blots.pdf]
